# Outbreak of *Achromobacter xylosoxidans* and *Ochrobactrum anthropi* Infections after Prostate Biopsies, France, 2014

**DOI:** 10.3201/eid2208.151423

**Published:** 2016-08

**Authors:** Skerdi Haviari, Pierre Cassier, Cédric Dananché, Monique Hulin, Olivier Dauwalder, Olivier Rouvière, Xavier Bertrand, Michel Perraud, Thomas Bénet, Philippe Vanhems

**Affiliations:** Hospices Civils de Lyon, Lyon, France (S. Haviari, P. Cassier, C. Dananché, M. Hulin, O. Dauwalder, O. Rouvière, M. Perraud, T. Bénet, P. Vanhems);; Université Claude Bernard, Lyon (S. Haviari, P. Cassier, C. Dananché, O. Dauwalder, O. Rouvière, M. Perraud, T. Bénet, P. Vanhems);; Centre International de Recherche en Infectiologie, Lyon (P. Cassier, O. Dauwalder, T. Bénet, P. Vanhems);; Centre Hospitalier Régional Universitaire de Besançon, Besançon, France (X. Bertrand);; Laboratoire des Pathogènes Émergents–Fondation Mérieux, Lyon (T. Bénet, P. Vanhems)

**Keywords:** Achromobacter xylosoxidans, Achromobacter denitrificans, Ochrobactrum anthropi, disease outbreaks, prostate, prostatitis, prostatic diseases, diagnostic techniques, clinical laboratory techniques, surgical, biopsy, gram-negative bacteria, bacteria, France

## Abstract

We report an outbreak of healthcare-associated prostatitis involving rare environmental pathogens in immunocompetent patients undergoing transrectal prostate biopsies at Hôpital Édouard Herriot (Lyon, France) during August 13–October 10, 2014. Despite a fluoroquinolone-based prophylaxis, 5 patients were infected with *Achromobacter xylosoxidans* and 3 with *Ochrobactrum anthropi*, which has not been reported as pathogenic in nonimmunocompromised persons. All patients recovered fully. Analysis of the outbreak included case investigation, case–control study, biopsy procedure review, microbiologic testing of environmental and clinical samples, and retrospective review of hospital records for 4 years before the outbreak. The cases resulted from asepsis errors during preparation of materials for the biopsies. A low-level outbreak involving environmental bacteria was likely present for years, masked by antimicrobial drug prophylaxis and a low number of cases. Healthcare personnel should promptly report unusual pathogens in immunocompetent patients to infection control units, and guidelines should explicitly mention asepsis during materials preparation.

## Abstract 2

Multiple cases of prostatitis resulted from improper infection control measures.

The diagnosis of prostate cancer relies heavily on transrectal ultrasound-guided prostate biopsy (TUPB), which 0.1%–0.3% of the total population undergoes each year in developed countries. An estimated 1 million biopsies are performed annually in the United States (*1*) and ≈63,000 in France (*2*). This invasive practice, essential to diagnose prostate cancer properly and to guide future treatment, takes several prostate samples by means of a biopsy needle, which passes through the intestinal barrier. This process makes proper asepsis challenging, and the attack rate of iatrogenic urinary tract infections (UTIs) after biopsy is ≈3%, although rates vary for different countries and clinics (*3*). Endogenous gram-negative bacteria, mostly *Escherichia coli*, are the main causative agents of complications after prostate biopsies (*4*). Antimicrobial drug prophylaxis is recommended for patients undergoing these procedures, mostly to reduce risk of infection. However, the choice of antimicrobial drug is always a compromise because a single drug cannot target all microorganisms (*5*–*7*). Fluoroquinolones targeting digestive gram-negative bacteria are the most common choice (*3*,*8*,*9*), as described in relevant guidelines (*10*,*11*).

Reports of a few outbreaks resulting from nonsterile handling of materials or inadequate procedures (*12*–*14*) have involved unexpected pathogens, such as naturally occurring environmental bacteria that are antimicrobial-drug resistant. These pathogens’ resistance to antimicrobial prophylaxis could theoretically facilitate outbreaks caused by asepsis errors. We investigated an outbreak of healthcare-associated UTIs occurring after prostate biopsies to stop its spread and determine its causes and risk factors.

## Materials and Methods

### Outbreak Description and Setting

In October 2014, the urology team of Hôpital Édouard Herriot, a teaching hospital with ≈850 beds, located in Lyon, France, alerted the radiology department that in the previous 3 weeks, 6 patients had dysuria and UTIs involving unusual pathogens <10 days after each had a prostate biopsy. The radiology department, which performed the biopsies, asked the hospital infection control unit to investigate, and the investigation began immediately.

Of the 6 initial case-patients, 4 had *Achromobacter xylosoxidans* UTIs and 2 had *Ochrobactrum anthropi* UTIs, all occurring after a TUPB. The radiology department performs ≈450 biopsies per year, all in the same ultrasound room. According to hospital records, ≈1% of patients call back to report symptoms of infections and need to submit urine or blood cultures within 15 days after biopsy.

### Biopsy Protocol

**Figure 1:**
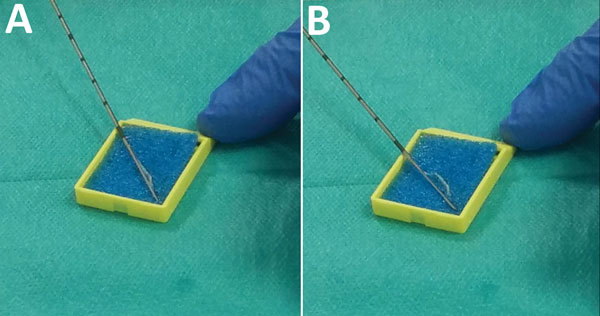
Application of biopsy needle on sponge used during transrectal ultrasound-guided prostate biopsy. A) Needle before scraping a biopsy core on sponge; B) needle after scraping to detach a biopsy core on sponge.

All biopsies were preceded by a single dose of antimicrobial drug prophylaxis (400 mg ofloxacin), as recommended by national guidelines at the time of the procedures (*11*). As a preliminary step, nonsterile sponges were soaked in a 0.9% solution of sodium chloride (NaCl) and placed in small plastic cases on a table beside the patient. During the first part of the procedure, the main operator undertook transrectal ultrasonography with a probe covered by an inner layer of gel, a protective plastic sheath, and an outer layer of gel. During the second part of the procedure, a needle guide was mounted on top of the plastic sheath, and the operator worked the automatic biopsy gun with his or her main hand, while holding the ultrasound probe with the other hand. After a sample was taken, the needle was scraped against one of the sponges to remove the biopsy core (Figure 1, panels A, B); the needle was then reused until all relevant samples were obtained. At the end of the procedure, the integrity of the probe sheath was checked, and all equipment was discarded or sanitized. Patients were advised to call their radiologist or urologist if they had fever, pain, difficulty urinating, or prolonged bleeding.

### Case–Control Study and Retrospective Surveillance

Administrative, clinical, and microbiologic records of all patients who underwent a TUPB at the hospital during September 1–October 31, 2014, were reviewed to find putative additional cases. The urology team was also asked to report any additional cases found as a result of systematic postbiopsy follow-up consultation. Confirmed cases were defined as patients having fever, pain, or dysuria and a positive urine or blood culture requested <15 days after biopsy that showed presence of *A. xylosoxidans* or *O. anthropi* infection. Controls were all patients who underwent a TUPB in the same ultrasound room during the same 2-month period but who were not asked to submit a blood or urine culture. Possible cases were retrospectively defined as patients for whom a clinician had requested >1 urine or blood culture <15 days after biopsy, regardless of the result, because of a clinical suspicion of prostatis (i.e., presence of fever, pain, or dysuria). Possible cases were not included in our statistical calculations.

Temporal distribution of cases involved an index case that occurred in August 2014, a month before the other cases occurred in September and October 2014 at a rate of ≈2 per week. All possible control-patients (n = 44) whose biopsies occurred between the first and last case of the September–October cluster were included. In addition, 6 temporally matched control-patients were included for the August case-patient; these patients were biopsied on the same day or <1 week before the case-patient.

Records were reviewed to assess organizational risk factors (e.g., operator identity or order in which patients had biopsies during the day) and individual features (e.g., patient age or histologic evidence of localized inflammation). Retrospective surveillance was also conducted by accessing an electronic database that contained dates and patient names for all prostate biopsies in the radiology unit and that contained dates, patient names, and results (i.e., species and antibiograms) for all blood and urine cultures in the hospital network laboratory during January 2011–November 2014. Records for November 2014–March 2015 were similarly analyzed to confirm the clinician-reported absence of new cases after implementation of corrective measures.

### Microbiologic Methods and Molecular Typing

All biopsy materials were investigated microbiologically. Tap water was gathered in sterile containers (Dominique Dutscher, Brumath, France). Sterile cotton swabs (Biolys, Taluyers, France) were used for transducer probe and sink drain samples. Worktop and handwashing sink samples were plated on Count Tact Agar plates (bioMérieux, Marcy l’Etoile, France). For *A. xylosoxidans* and *O. anthropi*, trypticase soy broth (TSB) was used as preenrichment broth, supplemented with cetrimide (2 g/L) for *A. xylosoxidans* and amoxicillin (10 mg/L) for *O. anthropi*. Brain–heart infusion (BHI) enrichment broth, supplemented with aztreonam (32 mg/L) and vancomycin (32 mg/L), was also used for both bacteria, as described (*15*).

For each species, 200 mL of tap water and 0.9% sterile NaCl were filtered through sterile 0.45-μm membrane filters (Millipore, Darmstadt, Germany). The membrane was placed either on plate count agar enriched with cetrimide (2 g/L) or on trypticase soy agar (TSA) enriched with amoxicillin (10 mg/L). Sponges, reused soaking container, probe, gel, plastic cases, and drain swab samples were first enriched with 10 mL of TSB supplemented with either cetrimide or amoxicillin for 48 h at 30°C or with BHI supplemented with aztreonam for 72 h at 37°C. One drop of each enrichment culture (i.e., TSB and centrimide, TSB and amoxicillin, and BHI and aztreonam) was respectively plated on TSA supplemented with cetrimide; on TSA supplemented with amoxicillin and incubated for 48 h at 30°C; and on MacConkey agar and incubated for 48 h at 37°C. Count Tact Agar plates were incubated for 7 days at 30°C.

Isolates were identified phenotypically by API 20NE and API 20E strips (bioMérieux) for non-*Enterobacteriacae* and *Enterobacteriacae*, respectively. Identification was confirmed by matrix-assisted laser desorption/ionization time-of-flight mass spectrometry with the VITEK MS system (bioMérieux).

The macrorestriction profile of the total DNA of the clinical and environmental isolates was charted by pulsed-field gel electrophoresis (PFGE) (CHEF-DR III, Bio-Rad, Hercules, California), as described (*16*). *Xba*I and *Spe*I served as restriction enzymes for *A. xylosoxidans* and *O. anthropi*, respectively. We ensured that the gels were comparable by including *Staphylococcus aureus* strain NCTC 8325 (including *Sma*I as the restriction enzyme) for reference. We compared PFGE patterns by visual inspection.

### Statistical Analysis

Descriptive statistics, including median and interquartile ranges, were computed for quantitative variables. Categorical data were compared by using the Fisher exact test and quantitative data by using the Mann-Whitney U test. Statistical analysis was conducted with R statistical software (*17*). All tests were 2-tailed, and p values <0.05 were considered statistically significant. Logistic regression was performed with general linear models with bias reduction as implemented in R, brglm (*18*,*19*). Multiple logistic regression that used an entry threshold of p = 0.15 and a backward elimination cutoff of p = 0.05 searched for factors that correlated independently with infection. Interactions between variables in the resulting model were also tested.

## Results

### Clinical Course

We identified 8 confirmed case-patients: 1 was biopsied on August 13, 2014; the other 7 were biopsied during September 12–October 10 and were evenly distributed during that period. An additional 2 possible case-patients with no microbiologic evidence of infection were biopsied on October 6 and 7 (Table 1). On the basis of confirmed cases, the attack rate during August 13–October 10 was 9.4% (8/85 patients). At the height of the outbreak (i.e., September 12–October 10), the rate was 13.2% (7/53). These rates compare with a baseline long-term rate of 1.4% (27/1,927 patients) for endogenous postbiopsy infections, which are defined as cultures collected <15 days postbiopsy and growing no environmental bacterial strains (determined from hospital records for January 2011–August 2014).

**Table 1:** Clinical and microbiologic history of the 8 confirmed cases in the cluster of *Achromobacter xylosoxidans* and *Ochrobactrum anthropi* infections occurring after prostate biopsies, France, 2014*

Characteristic	Patient ID							
	1	2	3	4	5	6	7	8
Date of biopsy	Aug 13	Sep 12	Sep 15	Sep 30	Sep 30	Oct 3	Oct 8	Oct 10
Patient age at onset, y	66	60	59	62	65	54	68	69
Days between biopsy and urinalysis	13	2	3	10	3	6	8	7
Highest fever level, °C	39	39	38.5	38.6	39.1	NR	39	38.3
Localized symptoms	No	Yes	No	Yes	No	NR	Yes	No
Hospitalization	Yes	Yes	Yes	Yes	Yes	No	No	No
Urine culture result	Negative	*A. xyl*	*A. xyl*	*A. xyl*	*A. xyl*	*O. ant*	*A. xyl*	*O. ant*
Blood culture result	*O. ant*	*A. xyl*	*A. xyl*	NA	*A. xyl*	NA	NA	NA
Curative antimicrobial treatment†	Yes	Yes	Yes	Yes	Yes	NR	Yes	No
Apyrexia without effective antimicrobial drug	Yes	No	Yes	No	Yes	NR	Yes	Yes
Immunosuppressive drugs	Yes	No	No	No	No	No	No	No

*A. xyl, *Achromobacter xylosoxidans*; NA, cultures not requested; NR, not recorded; *O. ant*, *Ochrobactrum anthropi.* †All patients received a single dose of 400 mg ofloxacin. Most received curative treatments: Patient 1, 2 g ceftriaxone/d intravenously for 3 d; then 200 mg ofloxacin/d orally for 10 d. Patient 2, ceftazidime, modalities unknown (treated outside the university hospital network). Patient 3, 2 g ceftriaxone 1× intravenously; then 200 mg ofloxacin 2×/d orally for 15 d. Patient 4, 2 g ceftriaxone/d intravenously for 3 d; then 1,200 mg amikacin 1× intravenously; then 800/160 mg co-trimoxazole 2×/d orally for 15 d. Patient 5, 2 g ceftriaxone/d intravenously for 3 d; then 4 g piperacillin 3×/d intravenously and 800/160 mg co-trimoxazole 3×/d orally for 4 d; then co-trimoxazole for 15 d. Patient 7, 1 g ceftriaxone/d intravenously for 2 d and 200 mg ofloxacin 2×/d orally for 21 d. Antibiogram of *A. xylosoxidans* for patients 2, 3, 4, 5, and 7 showed sensitivity to amoxicillin, ticarcillin, piperacillin (with or without β-lactamase inhibitors), ceftazidime, colistin, co-trimoxazole, and carbapenems and resistance to cefalotine, cefoxitine, cefotaxime, cefepime, aminoglycosides, quinolones, tigecyclin, fosfomycin, and rifampin. Antibiogram of *O. anthropi* for patient 1 showed sensitivity to carbapenems, aminoglycosides, ciprofloxacin, tigecyclin, rifampin, and co-trimoxazole and resistance to amoxicillin, ticarcillin, piperacillin (with or without β-lactamase inhibitors), cefalotine, cefoxitine, cefotaxime, ceftazidime, cefepime, aztreonam, norfloxacin, and fosfomycin. Antibiogram of *O. anthropi* for patient 8 was the same as for patient 1 except for sensitivity to norfloxacin. In hindsight, co-trimoxazole should probably have been used as first-line therapy.

Of the 10 confirmed and possible case-patients, 6 were hospitalized. None were in intensive care, and all recovered fully. Five patients were infected with an *A. xylosoxidans* strain resistant to ceftriaxone and ofloxacin; 3 of these patients reached apyrexia within 48 hours of treatment initiation with ceftriaxone (2 g/24 h), ofloxacin (400 mg/24 h), or both, as first-line regimens, despite the in vitro resistance to these agents. Three patients had *O. anthropi* infection; 1 received no curative antimicrobial drug treatment. The first outbreak patient (biopsied on August 13) had *O. anthropi* bacteremia and was a kidney-transplant recipient. The 2 possible case-patients with no identifiable culture also recovered quickly.

### Onsite Investigation

Facility inspection and a review of practices identified mistakes in the sterile handling of sponges, which were sometimes accidentally touched by an aide with contaminated hands (aide 1 in Table 2). The container in which the sponges were soaked was reused from day to day, reportedly for up to several weeks. The container was commonly left overnight with sponges inside and never completely dried during the course of use. Other practices causing risk included application of nonsterile gel on the outer layer of the ultrasound probe and lack of a proper log of probe disinfection procedures.

**Table 2:** Characteristics and risk factors of patients and controls in the study of an outbreak of *Achromobacter xylosoxidans* and *Ochrobactrum anthropi* infections occurring after prostate biopsies, France, 2014*

Variables	Cases, n = 8	Controls, n = 50	p value	Crude OR (95% CI)	Adjusted OR (95% CI)
Quantitative variables, median (IQR)†					
Age, y	63.5 (59.6–67.2)	67.1 (60.4–73.7)	0.12	0.55 (0.180–1.43)‡	–
Prostate-specific antigen, µg/L	6.94 (6.22–8.5)	6.0 (4.61–8.0)	0.3	1.36 (0.429–3.39)‡	–
Prostate size, mL§	40.0 (30.5–54.0)	37.2 (26.2–58.8)	0.39	1.28 (0.680–2.37)‡	–
No. cores sampled	14.5 (12.8–15.2)	15.0 (13.2–15.8)	0.92	0.97 (0.694–1.20)‡	–

Categorical variables, no. (%)¶					
Organizational factors					
Previous unaffected patient that day#	0	31 (62)	0.001	0.04 (0.000–0.319)	–
First patient of the day	7 (87.5)	14 (28)	0.002	12.6 2.44–353)	14.5 (2.49–558)
Operator A	5 (62.5)	9 (18)	0.02	6.86 1.56–43.0)	8.49 (1.55–104)
Aide 1 alone	7 (87.5)	27 (54)	0.12	4.27 (0.844–116)	–

Physiologic factors					
Previously had biopsy	4 (50)	41 (82)	0.07	0.23 (0.044–1.08)	–
Inflammatory histology	2 (25)	5 (10)	0.24	3.18 (0.373–17.9)	–
Previous localized treatment**	0	10 (20)	0.33	0.23 (0.000–2.09)	–
Neoplasic histology	5 (62.5)	41 (82)	0.34	0.36 (0.075–2.04)	–

*HIFU, high-intensity focused ultrasound; OR, odds ratio; –, not included in the final multiple regression model.  †p values determined by using the Mann-Whitney U test.  ‡For every additional 10 y in age, 5 µg/L (prostate-specific antigen), 20 mL (prostate size), and additional core (number of cores). §n = 6 for cases, n = 44 for controls. ¶p values determined by using Fisher exact test.  #This variable was not included in the multiple model because it was closely correlated wtih the variable “first patient of the day.” **Resection or HIFU.

The plastic container was identified as a likely source of environmental contamination of the biopsy needle, with bacteria being spread by the sponges. The container was immediately taken for microbiologic examination and replaced by sterile single-use cups. Biopsies continued to be performed while the investigation continued.

Samples were also collected from the sterile saline flask periodically used to refill the container and from the plastic cases; sponges (dry and soaked); ultrasound gel; transducer probe; tap water; and biopsy room environment (i.e., worktop, sink, and drain). A total of 52 samples were collected from 20 soaked sponges, 6 dry sponges, 2 plastic cases, 1 NaCl 0.9% flask, 1 ultrasound gel bottle, 4 water samples, and 18 surfaces.

### Microbiologic Studies and Molecular Typing

**Figure 2:**
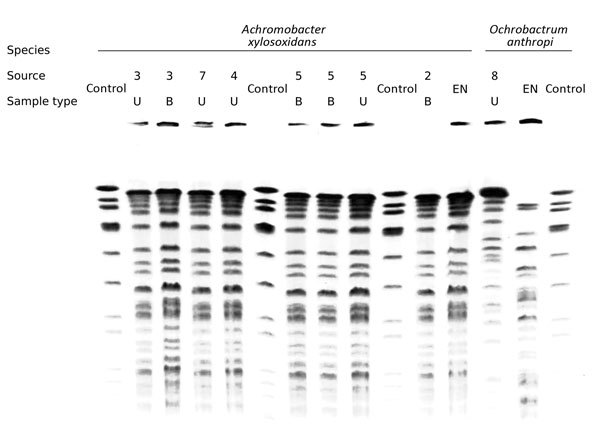
Pulsed-field gel electrophoresis of clinical and environmental strains of *Achromobacter xylosoxidans* and *Ochrobactrum anthropi* infections in patients after undergoing prostate biopsies at Hôpital Édouard Herriot, Lyon, France. Patient numbers match those in Table 1. EN, environmental; B, blood; U, urine; control, reference sample for calibration (described in Methods).

All samples tested negative except for sponges retrieved from the saline-filled plastic container; those samples tested positive for 5 bacterial species: *A. xylosoxidans*, *O. anthropi*, *Stenotrophomonas maltophilia, Roseomonas mucosa*, and *Enterobacter cloacae*. Neither the dry sponges nor the sterile NaCl 0.9% flask used to soak them was contaminated. Of the 5 bacterial isolates, only *A. xylosoxidans* was resistant to fluoroquinolones. PFGE confirmed that all 5 patients infected by *A. xylosoxidans* were contaminated by the same strain (Figure 2). However, the clinical and environmental *O. anthropi* isolates had different PFGE profiles.

### Infection Control Measures and Outbreak Termination

On the day of the alert, investigators conducting facility inspection identified the plastic container as a potential hazard and removed it from use. Beginning the following morning, the sponges were soaked in sterile cups, and a new cup was used for each patient. In early 2015, all sponges were systematically sterilized before being placed for soaking in sterile single-use cups. Radiology staff were retrained in clean and sterile handling of materials. No microbiologically confirmed infections occurred during October 10, 2014–March 30, 2015.

### Case–Control Study

Case–control investigation determined that among potential risk factors considered (Table 2), first procedure of the day and identity of the main operator were associated with increased infection risk (adjusted odds ratio 14.5 [95% CI 2.49–558] and 8.49 [95% CI 1.55–104], respectively). No other significant differences between case-patients and control-patients were apparent; an interaction term variable was also considered and was not statistically significant. A sensitivity analysis, whether it included possible cases or excluded August cases and controls, retained the same variables in the final model (data not shown).

### Retrospective Surveillance

After being informed by radiology staff of prolonged sporadic reuse of the plastic containers, we reviewed databases for January 2011–August 2014 for TUPBs and blood or urine cultures, looking for past cases involving 1 of the 5 species isolated from the container or other rare, environmental, waterborne pathogens. For 32 (1.7%) of 1,927 patients undergoing the procedure, a blood or urine sample was taken <15 days after the biopsy. Overall, cultures for 23 of the 32 patients were negative; among the other 9 patients, 2 had positive urine cultures with >2 bacteria (suggesting contamination by digestive flora), 1 had *Klebsiella pneumoniae*, 1 had *E. coli*, 1 had *O. anthropi* (patient 1 from Table 1), and 4 had *S. maltophilia* (1 of the 5 species found in the container) (Table 3).

**Table 3:** Clinical and microbiologic history of retrospective surveillance patients in the study of an outbreak of *Achromobacter xylosoxidans* and *Ochrobactrum anthropi* infections occurring after prostate biopsies, France*

Characteristic	Patient ID					
	A	B	C	D	E	F
Date of biopsy	2011 Nov 23	2011 Dec 16	2012 May 7	2012 May 31	2012 July 9	2012 Nov 30
Patient age at onset, y	57	79	75	65	76	68
Days between biopsy and urinalysis	5	1	8	3	9	2
Highest fever, °C	40	39	None	39	38.4	None
Localized symptoms	Yes	Yes	Yes	No	Yes	Yes
Hospitalization	No	Yes	No	No	No	No
Urine culture result	*S. malt*	*S. malt*	*E. coli*	*S. malt*	*S. malt*	Mixed flora
Blood culture result	NA	Negative	NA	Negative	NA	*K. pne*
Antimicrobial resistance profile†	1	1	NR	2	3	NR
Curative antimicrobial drug treatment	Yes	Yes	Yes	Yes	Yes	Yes
Apyrexia without proper antimicrobial regimen	Yes	Yes	No	Yes	Yes	Yes
Immunosuppressants	No	No	No	No	No	No
*E. coli, *Escherichia coli;* *K. pne*, *Klebsiella pneumoniae;* NA, cultures not requested; NR, not recorded; *S. malt*, *Stenotrophomonas maltophilia*. †The antimicrobial-drug resistance profile is shown for *S. maltophilia* only. Profiles 1 and 2 had only 1 difference across 17 different antimicrobial drugs (ceftazidime-I vs. -S). Of 14 antimicrobial drugs tested for all 4 *S. maltophilia* patients, profile 3 had 2 differences from profile 2 and 3 differences from profile 1.						

During 2011 and 2012, four *S. maltophilia* cases clustered in 2 pairs, for which TUPBs occurred 19 days and 45 days apart. The first pair of patients had identical antibiograms, indicating that they were likely infected by the same strain. Minor differences were found in the antibiograms of the second pair. All strains were resistant to fluoroquinolones and third-generation cephalosporins. No complications occurred. All 4 patients were the first to undergo a biopsy on the day of their procedure.

## Discussion

Our investigation showed that an outbreak of infections with uncommon pathogens after TUPBs in a radiology clinic resulted from inadequate preparation of supplies for the procedure. An aide seems to have accidentally contaminated a container, and its prolonged reuse for soaking small sponges enabled proliferation of uncommon waterborne pathogens and contamination of the sponges. For each patient, the biopsy needle was repeatedly in contact with contaminated sponges (Figure 1, panels A, B) and was reused to obtain additional biopsy cores, inoculating some patients with pathogens and causing infections. Because an ultrasound gel contaminated with *A. xylosoxidans* had been previously linked to an outbreak (*12*), we tested both unopened and opened bottles of gel from the radiology clinic; all results were negative. Other outbreaks involving *A. xylosoxidans* have been reported (*12*,*20*–*23*) and sometimes attributed to this environmental organism’s ability to colonize reusable plastic containers (*20*,*21*). Its resistance to common disinfectants, especially quaternary ammonium compounds (*20*,*21*,*24*), makes single-use equipment all the more critical.

We found other reports suggesting particular prostate susceptibility to this type of organism. One report involved this species after prostate biopsies (*12*); others involved the same organ and other lung pathogens classically associated with cystic fibrosis, such as *Pseudomonas aeruginosa* and *Burkholderia cepacia* (*13*,*14*). We found 3 *O. anthropi–*related outbreaks reported in the literature; all involved specific patient populations that received immunosuppressants systemically (*25*,*26*) or locally (*27*). In contrast, 2 of the 3 patients infected with *O. anthropi* described in our study were immunocompetent.

**Figure 3:**
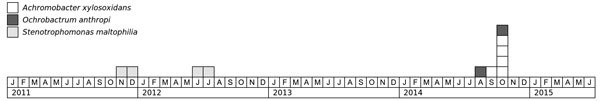
Patients with positive urine or blood cultures taken <15 days after prostate biopsy at Hôpital Édouard Herriot, Lyon, France, 2011–2015. Only nondigestive species matching those found in a contaminated pot in 2014 are shown. All represented patients are from hospital data files. For consistency, 1 patient with *Ochrobactrum* *anthropi* infection treated in primary care in October 2014 is not shown.

In the outbreak we report, 5 cases were caused by *A. xylosoxidans*, a waterborne opportunistic pathogen that rarely causes clinically relevant infections. The same strain was responsible for all 5 of these infections and was fluoroquinolone resistant, enabling the strain to escape prophylaxis and facilitating the outbreak (Figure 3).

The other species, *O. anthropi*, was the source of infection for 3 case-patients. This pathogen caused symptomatic infections despite its susceptibility to antimicrobial drug prophylaxis (ofloxacin). Our study’s retrospective nature resulted in our inability to ascertain definitely whether prophylaxis had been taken properly or whether this strain was capable of surviving despite its in vitro susceptibility. Because the contamination of the plastic container was extensive and because a PFGE mismatch occurred (Figure 2), other hypotheses for the proliferation of the strain include the possibilities that a large inoculum of the pathogen occurred or that several strains of this species were involved in these infections.

No pathogen could be identified in 2 possible cases occurring during the outbreak period. These patients might correspond to either false positives or to real cases despite having negative cultures because of prophylaxis (i.e., 4 of 5 strains isolated from the container were sensitive to fluoroquinolones); these cases’ occurrence during the time of the other cases argues for the latter hypothesis. We excluded these 2 cases from the case–control analysis because their status could not be ascertained.

We also found 4 cases of infection with *S. maltophilia*, another pathogen associated with stagnant water, from 2011–2012. At that time, radiology staff members were reusing plastic containers over a period of several days or weeks. We cannot attribute these past infections to similar contamination with certainty. However, postbiopsy infections with *S. maltophilia* are nearly nonexistent in the literature (*4*,*28*), and an isolate of this species was retrieved from environmental contamination during the 2014 outbreak. The clinical isolates from 2011–2012 were resistant to antimicrobial drug prophylaxis (ofloxacin) in vitro, whereas the environmental isolate from 2014 was not; this change in susceptibility could explain why *S. maltophilia* infections occurred in 2011–2012 but not in 2014. The same type of mishandling of materials, although with less severe contamination, was likely responsible for the *S. maltophilia* cases occurring in 2011–2012. These cases were overlooked, probably because no sizeable cluster occurred, unlike in 2014.

During prostate biopsy, all items that enter sterile tissue are critical and should be sterile, along with items that will not be in contact with patients but will be in contact with critical items, as discussed in national recommendations (*11*) and elsewhere (*29*). Generally, all reusable items should be cleaned or sterilized (*30*). Although the nonsterile nature of the rectum and antimicrobial drug prophylaxis might result in healthcare workers’ being less vigilant in following recommendations, outbreaks caused by prophylaxis-resistant environmental bacteria indicate the need for rigorous implementation of sterile practices in all settings. We found no specific mention of the need for such materials to be sterile in various recommendations (*30*–*33*), with the exception of an annex in French national guidelines (*11*). This omission in recommendations might contribute to oversights such as those reported in this article, especially given the frequency of the procedure. After the outbreak, practices were modified and now comply fully with French guidelines.

Other preventive measures have been discussed in the literature. Some are specifically aimed at endogenous bacteria, such as the use of a transperineal route (*34*) or prebiopsy rectal cultures to guide antimicrobial-drug prophylaxis (*9*); others are more general, such as providone/iodine enemas (*9*) and formalin disinfection of biopsy needles (*35*). Such preventive measures have had good preliminary results and could possibly have prevented this outbreak.

This outbreak highlights the usefulness of analyzing the order of procedures to detect environmental contamination. In our investigation, the first patient of the day had a higher infection risk than subsequent patients. The bacterial inoculum was likely highest for the first procedure of the day and then decreased for remaining patients because the highly contaminated sponges, which often soaked overnight, had been used and new ones were soaked only briefly in the plastic container.

Some limitations reduce the strength of our study. The urgent need to terminate the outbreak made the data observational, the sample size low, and the study mostly retrospective and subject to recording and reporting bias. Also, the possibility exists of limited sensitivity of a diagnosis that is based on voluntary consultation, especially given that the UTIs were generally mild and, for some patients, symptoms might have been present and worrying but were overlooked by physicians inside or outside the university hospital, contributing to a misclassification bias. However, this possible bias might be limited because little time had passed between clinical onset of cases and the investigation and because all patients had to consult with urologists to obtain their biopsy results. Previous cases involving other bacteria might have escaped our analysis because of antimicrobial drug prophylaxis, which likely biased the microbiologic results for some patients, and because no cluster of cases existed to draw increased scrutiny from clinical teams.

In summary, an outbreak of healthcare-associated infections after TUPBs was caused by improper handling of biopsy materials and involved 2 unusual pathogens, *O. anthropi* and *A. xylosoxidans*. The latter was resistant to standard fluoroquinolone-based antimicrobial drug prophylaxis. Infections by uncommon microorganisms should quickly be reported to infection control units, especially when they cluster in time. Invasive outpatient procedures, such as TUPBs, could be the source of other outbreaks involving multidrug-resistant environmental bacteria. Such procedures require rigorous sterile handling of all relevant materials during operating room surgery.
